# Editorial Notes

**Published:** 1937

**Authors:** 


### Editorial Notes

**Central Health Clinic and Ham Green Extensions.**

The opening of the Central Health Clinic on July 9th by Sir Farquhar Buzzard, Regius Professor of Medicine at Oxford, and of the Ham Green Hospital Extensions on September 23rd by Professor W. W. Jameson, mark two notable additions to the equipment of the health services of our city. In the Central Health Clinic accommodation is provided for doctors, health visitors, sanitary inspectors and clerks. Infant welfare, ante-natal and dental clinics are established there. The tuberculosis officer will conduct the main work of the tuberculosis dispensary in the central clinic; it will be the headquarters of school medical inspection and of certain specialist medical services. There will also be facilities for general health education. The building is designed with a view to rapid and easy inter-communication between the various departments, and there can be no doubt that it will be of the greatest value to the inhabitants of Bristol. It is, in fact, one of the first steps towards the realization of the Health Campaign launched by the Prime Minister in London on Thursday, 30th September. Unfortunately the building does not give the impression of being designed in accordance with the most modern notions, and it is questionable whether the internal planning of rooms and corridors will prove entirely satisfactory. There is something old-fashioned about the general conception of the building which becomes very noticeable when contrasted with the extensions at Ham Green Hospital. Perhaps it would be more correct to say "when contrasted with the 'cubicle type' ward blocks intended for the treatment of general infectious cases when small-pox is absent." These blocks with their wide verandahs are, without question, the best examples of ward construction in Bristol, or perhaps in Great Britain as a whole. The Health Committee, the Medical Officer of Health, and the Architect deserve the highest praise for the excellence of these blocks. The 14-bed block which is to be kept always ready for the reception of small-pox cases only is a poor affair in comparison, for in this block the cubicle system has not been adopted. The wards are well-built, pleasantly decorated and have a homely, antiquated appearance. Professor Jameson's address (see p. 221) was so flattering to the spirit of progress existing in Bristol that it is hard to resist the temptation to speculate on the relative responsibilities of our own Health Committee and the Ministry of Health in the planning of the Central Health Clinic and the Ham Green extensions.

**Dr. Beddoes and the Physical Fitness Campaign.**

THE modern devotion to the problem of keeping fit is not quite so modern as is popularly supposed. It puzzled Dr. Beddoes, the famous director of the Clifton Pneumatic Institute. He expressed his views in the uncompromising manner that lost him his professional chair at Oxford. "Task-exercises, in my opinion, hold the same proportion to health as the castigations of penitents to piety and virtue." Another explosion of his reads, "The dumb-bell I consider as a miserable substitute for running, digging, working with tools and those other exertions that engage the mind, while they put the limbs into motion. Curious sports, such as archery, have not a permanent interest." Evidently there were irreverent spirits abroad even before Geoffrey Gorer wrote in *Africa Dances*: "The whole of English life in West Africa revolves very properly round the problem of keeping fit. It is, of course, axiomatic that you can only keep fit on pieces of ground specially devoted to that end; there is no virtue in movement over soil which is also employed for utilitarian purposes." But Milton, as usual, finds the stinging word to combine with his rare wisdom when he refers scornfully to the "empty and unrecruitable colonels of twenty men in a company" whom he regarded as useless for the education of the young in healthy exercises. His view was that "in those vernal seasons of the year when the air is calm and pleasant, it were an injury and sullenness against nature, not to go out and see her riches, and partake in her rejoicing with heaven and earth." Even Mr. Osbert Sitwell would be hard pressed to improve on that phrase, "Empty and unrecruitable colonels"!

